# The ARRIVE guidelines 2.0: updated guidelines for reporting animal research

**DOI:** 10.1136/bmjos-2020-100115

**Published:** 2020-07-20

**Authors:** Nathalie Percie du Sert, Viki Hurst, Amrita Ahluwalia, Sabina Alam, Marc T Avey, Monya Baker, William J Browne, Alejandra Clark, Innes C Cuthill, Ulrich Dirnagl, Michael Emerson, Paul Garner, Stephen T Holgate, David W Howells, Natasha A Karp, Stanley E Lazic, Katie Lidster, Catriona J MacCallum, Malcolm Macleod, Esther J Pearl, Ole H Petersen, Frances Rawle, Penny Reynolds, Kieron Rooney, Emily S Sena, Shai D Silberberg, Thomas Steckler, Hanno Wuerbel

**Affiliations:** 1 Experimental Design and Reporting, NC3Rs, London, UK; 2 William Harvey Research Institute, London, UK; 3 Queen Mary University of London, London, UK; 4 Taylor & Francis Group, London, UK; 5 ICF, Durham, North Carolina, USA; 6 Opinion, Nature, San Francisco, California, USA; 7 School of Education, University of Bristol, Bristol, UK; 8 Life Sciences, PLOS ONE, Cambridge, UK; 9 School of Biological Sciences, University of Bristol, Bristol, UK; 10 Quest Center for Transforming Biomedical Research, Berlin Institute of Health, Berlin, Germany; 11 Department of Experimental Neurology, Charite Universitatsmedizin Berlin, Berlin, Germany; 12 National Heart and Lung Institute, Imperial College London, London, UK; 13 Centre for Evidence Synthesis in Global Health, Clinical Sciences Department, Liverpool School of Tropical Medicine, Liverpool, UK; 14 Clinical and Experimental Sciences, University of Southampton, Southampton, Hampshire, UK; 15 Tasmanian School of Medicine, University of Tasmania, Hobart, Tasmania, Australia; 16 Data Sciences & Quantitative Biology, Discovery Sciences, R&D, AstraZeneca PLC, Cambridge, Cambridgeshire, UK; 17 Prioris.ai, Ottawa, Ontario, Canada; 18 Animal Welfare, NC3Rs, London, UK; 19 Open Science, Hindawi Limited, London, UK; 20 Academic Lead for Research Improvement and Research Integrity, University of Edinburgh, Edinburgh, UK; 21 Centre for Clinical Brain Sciences, University of Edinburgh, Edinburgh, UK; 22 Academia Europaea Knowledge Hub, Cardiff University, Cardiff, South Glamorgan, UK; 23 Policy, Ethics and Governance, Medical Research Council, London, UK; 24 Department of Anesthesiology, University of Florida College of Medicine, Gainesville, Florida, USA; 25 Faculty of Medicine and Health, The University of Sydney, Sydney, New South Wales, Australia; 26 Research Quality, National Institute of Neurological Disorders and Stroke, Bethesda, Maryland, USA; 27 Janssen Pharmaceutica NV, Beerse, Belgium; 28 Veterinary Public Health Institute, Vetsuisse Faculty, University of Bern, Bern, Switzerland

**Keywords:** ARRIVE, reporting guidelines, animal research, reproducibility

## Abstract

Reproducible science requires transparent reporting. The ARRIVE guidelines (Animal Research: Reporting of In Vivo Experiments) were originally developed in 2010 to improve the reporting of animal research. They consist of a checklist of information to include in publications describing in vivo experiments to enable others to scrutinise the work adequately, evaluate its methodological rigour and reproduce the methods and results. Despite considerable levels of endorsement by funders and journals over the years, adherence to the guidelines has been inconsistent, and the anticipated improvements in the quality of reporting in animal research publications have not been achieved. Here, we introduce ARRIVE 2.0. The guidelines have been updated and information reorganised to facilitate their use in practice. We used a Delphi exercise to prioritise and divide the items of the guidelines into two sets, the ‘ARRIVE Essential 10’, which constitutes the minimum requirement, and the ‘Recommended Set’, which describes the research context. This division facilitates improved reporting of animal research by supporting a stepwise approach to implementation. This helps journal editors and reviewers verify that the most important items are being reported in manuscripts. We have also developed the accompanying Explanation and Elaboration document, which serves (1) to explain the rationale behind each item in the guidelines, (2) to clarify key concepts and (3) to provide illustrative examples. We aim, through these changes, to help ensure that researchers, reviewers and journal editors are better equipped to improve the rigour and transparency of the scientific process and thus reproducibility.

## Why good reporting is important

In recent years, concerns about the reproducibility of research findings have been raised by scientists, funders, research users and policy makers.^1 2^ Factors that contribute to poor reproducibility include flawed study design and analysis, variability and inadequate validation of reagents and other biological materials, insufficient reporting of methodology and results and barriers to accessing data.^3^ The bioscience community has introduced a range of initiatives to address the problem, from open access and open practices to enable the scrutiny of all aspects of the research^4 5^ through to study preregistration to shift the focus towards robust methods rather than the novelty of the results,^6 7^ as well as resources to improve experimental design and statistical analysis.^8–10^

Transparent reporting of research methods and findings is an essential component of reproducibility. Without this, the methodological rigour of the studies cannot be adequately scrutinised, the reliability of the findings cannot be assessed and the work cannot be repeated or built on by others. Despite the development of specific reporting guidelines for preclinical and clinical research, evidence suggests that scientific publications often lack key information and that there continues to be considerable scope for improvement.^11–18^ Animal research is a good case in point, where poor reporting impacts on the development of therapeutics and irreproducible findings can spawn an entire field of research, or trigger clinical studies, subjecting patients to interventions unlikely to be effective.^2 19 20^

In an attempt to improve the reporting of animal research, the ARRIVE guidelines (Animal Research: Reporting of In Vivo Experiments) were published in 2010. The guidelines consist of a checklist of the items that should be included in any manuscript that reports in vivo experiments, to ensure a comprehensive and transparent description.^21–30^ They apply to any area of research using live animal species and are especially pertinent to describe comparative research in the laboratory or other formal test setting. The guidelines are also relevant in a wider context, for example, for observational research, studies conducted in the field and where animal tissues are used. In the 10 years since publication, the ARRIVE guidelines have been endorsed by >1000 journals from across the life sciences. Endorsement typically includes advocating their use in guidance to authors and reviewers. However, despite this level of support, recent studies have shown that important information as set out in the ARRIVE guidelines is still missing from most publications sampled. This includes details on randomisation (reported in only 30%–40% of publications), blinding (reported in only approximately 20% of publications), sample size justification (reported in <10% of publications) and animal characteristics (all basic characteristics reported in <10% of publications).^11 31 32^

Evidence suggests that two main factors limit the impact of the ARRIVE guidelines. The first is the extent to which editorial and journal staff are actively involved in enforcing reporting standards. This is illustrated by a randomised controlled trial at *PLOS ONE*, designed to test the effect of requesting a completed ARRIVE checklist in the manuscript submission process. This single editorial intervention, which did not include further verification from journal staff, failed to improve the disclosure of information in published papers.^33^ In contrast, other studies using shorter checklists (primarily focused on experimental design) with more editorial follow-up have shown a marked improvement in the nature and detail of the information included in publications.^34–36^ It is likely that the level of resource required from journals and editors currently prohibits the implementation of all the items of the ARRIVE guidelines.

The second issue is that researchers and other individuals and organisations responsible for the integrity of the research process are not sufficiently aware of the consequences of incomplete reporting. There is some evidence that awareness of ARRIVE is linked to the use of more rigorous experimental design standards^37^; however, researchers are often unfamiliar with the much larger systemic bias in the publication of research and in the reliability of certain findings and even of entire fields.^33 38–40^ This lack of understanding affects how experiments are designed and grant proposals prepared, how animals are used and data recorded in the laboratory and how manuscripts are written by authors or assessed by journal staff, editors and reviewers.

Approval for experiments involving animals is generally based on a harm-benefit analysis, weighing the harms to the animals involved against the benefits of the research to society. If the research is not reported in enough detail, even when conducted rigorously, the benefits may not be realised, and the harm-benefit analysis and public trust in the research are undermined.^41^ As a community, we must do better to ensure that, where animals are used, the research is both well designed and analysed as well as transparently reported. Here, we introduce the revised ARRIVE guidelines, referred to as ARRIVE 2.0. The information included has been updated, extended and reorganised to facilitate the use of the guidelines, helping to ensure that researchers, editors and reviewers—as well as other relevant journal staff—are better equipped to improve the rigour and reproducibility of animal research.

## Introducing ARRIVE 2.0

In ARRIVE 2.0, we have improved the clarity of the guidelines, prioritised the items, added new information, and generated the accompanying Explanation and Elaboration (E&E) document to provide context and rationale for each item^42^ (also available at https://www.arriveguidelines.org). New additions comprise inclusion and exclusion criteria, which are a key aspect of data handling and prevent the ad hoc exclusion of data^43^; protocol registration, a recently emerged approach that promotes scientific rigour and encourages researchers to carefully consider the experimental design and analysis plan before any data are collected^44^; and data access, in line with the FAIR Data Principles (Findable, Accessible, Interoperable, Reusable).^45^ S1 Table summarises the changes.

The most significant departure from the original guidelines is the classification of items into two prioritised groups, as shown in tables 1 and 2. There is no ranking of the items within each group. The first group is the ‘ARRIVE Essential 10’, which describes information that is the basic minimum to include in a manuscript, as without this information, reviewers and readers cannot confidently assess the reliability of the findings presented. It includes details on the study design, the sample size, measures to reduce subjective bias, outcome measures, statistical methods, the animals, experimental procedures and results. The second group, referred to as the ‘Recommended Set’, adds context to the study described. This includes the ethical statement, declaration of interest, protocol registration and data access, as well as more detailed information on the methodology such as animal housing, husbandry, care and monitoring. Items on the abstract, background, objectives, interpretation and generalisability also describe what to include in the more narrative parts of a manuscript.

**Table 1:** ARRIVE Essential 10

ARRIVE Essential 10		
Study design	1	For each experiment, provide brief details of study design including: The groups being compared, including control groups. If no control group has been used, the rationale should be stated. The experimental unit (eg, a single animal, litter or cage of animals).
Sample size	2	Specify the exact number of experimental units allocated to each group, and the total number in each experiment. Also indicate the total number of animals used. Explain how the sample size was decided. Provide details of any a priori sample size calculation, if done.
Inclusion and exclusion criteria	3	Describe any criteria used for including and excluding animals (or experimental units) during the experiment, and data points during the analysis. Specify if these criteria were established a priori. If no criteria were set, state this explicitly. For each experimental group, report any animals, experimental units or data points not included in the analysis and explain why. If there were no exclusions, state so. For each analysis, report the exact value of *n* in each experimental group.
Randomisation	4	State whether randomisation was used to allocate experimental units to control and treatment groups. If done, provide the method used to generate the randomisation sequence. Describe the strategy used to minimise potential confounders such as the order of treatments and measurements, or animal/cage location. If confounders were not controlled, state this explicitly.
Blinding	5	Describe who was aware of the group allocation at the different stages of the experiment (during the allocation, the conduct of the experiment, the outcome assessment and the data analysis).
Outcome measures	6	Clearly define all outcome measures assessed (eg, cell death, molecular markers or behavioural changes). For hypothesis-testing studies, specify the primary outcome measure, ie, the outcome measure that was used to determine the sample size.
Statistical methods	7	Provide details of the statistical methods used for each analysis, including software used. Describe any methods used to assess whether the data met the assumptions of the statistical approach, and what was done if the assumptions were not met.
Experimental animals	8	Provide species-appropriate details of the animals used, including species, strain and substrain, sex, age or developmental stage and, if relevant, weight. Provide further relevant information on the provenance of animals, health/immune status, genetic modification status, genotype and any previous procedures.
Experimental procedures	9	For each experimental group, including controls, describe the procedures in enough detail to allow others to replicate them, including: What was done, how it was done and what was used. When and how often. Where (including detail of any acclimatisation periods). Why (provide rationale for procedures).
Results	10	For each experiment conducted, including independent replications, report: Summary/descriptive statistics for each experimental group, with a measure of variability where applicable (eg, mean and SD, or median and range). If applicable, the effect size with a confidence interval.

Explanations and examples for items 1–10 are available in the Explanation and Elaboration document^42^ and on the website at: https://www.arriveguidelines.org.

ARRIVE, Animal Research: Reporting of In Vivo Experiments.

**Table 2:** ARRIVE Recommended Set

Recommended Set		
Abstract	11	Provide an accurate summary of the research objectives, animal species, strain and sex, key methods, principal findings and study conclusions.
Background	12	Include sufficient scientific background to understand the rationale and context for the study, and explain the experimental approach. Explain how the animal species and model used address the scientific objectives and, where appropriate, the relevance to human biology.
Objectives	13	Clearly describe the research question, research objectives and, where appropriate, specific hypotheses being tested.
Ethical statement	14	Provide the name of the ethical review committee or equivalent that has approved the use of animals in this study, and any relevant licence or protocol numbers (if applicable). If ethical approval was not sought or granted, provide a justification.
Housing and husbandry	15	Provide details of housing and husbandry conditions, including any environmental enrichment.
Animal care and monitoring	16	Describe any interventions or steps taken in the experimental protocols to reduce pain, suffering and distress. Report any expected or unexpected adverse events. Describe the humane end points established for the study, the signs that were monitored and the frequency of monitoring. If the study did not have humane end points, state this.
Interpretation/Scientific implications	17	Interpret the results, taking into account the study objectives and hypotheses, current theory and other relevant studies in the literature. Comment on the study limitations, including potential sources of bias, limitations of the animal model and imprecision associated with the results.
Generalisability/Translation	18	Comment on whether, and how, the findings of this study are likely to generalise to other species or experimental conditions, including any relevance to human biology (where appropriate).
Protocol registration	19	Provide a statement indicating whether a protocol (including the research question, key design features and analysis plan) was prepared before the study, and if and where this protocol was registered.
Data access	20	Provide a statement describing if and where study data are available.
Declaration of interests	21	Declare any potential conflicts of interest, including financial and non-financial. If none exists, this should be stated. List all funding sources (including grant identifier) and the role of the funder(s) in the design, analysis and reporting of the study.

Together with the Essential 10, the Recommended Set represents best reporting practice. Explanations and examples for items 11–21 are available in the Explanation and Elaboration document^42^ and on the website https://www.arriveguidelines.org.

ARRIVE, Animal Research: Reporting of In Vivo Experiments.

Revising the guidelines has been an extensive and collaborative effort, with input from the scientific community carefully built into the process. The revision of the ARRIVE guidelines has been undertaken by an international working group—the authors of this publication—with expertise from across the life sciences community, including funders, journal editors, statisticians, methodologists and researchers from academia and industry. We used a Delphi exercise^46^ with external stakeholders to maximise diversity in fields of expertise and geographical location, with experts from 19 countries providing feedback on each item, suggesting new items and ranking items according to their relative importance for assessing the reliability of research findings. This ranking resulted in the prioritisation of the items of the guidelines into the two sets. Demographics of the Delphi panel and full methods and results are presented in Supporting Information S1 Delphi and S1 Data. Following their publication on BioRxiv, the revised guidelines and the E&E were also road tested with researchers preparing manuscripts describing in vivo studies, to ensure that these documents were well understood and useful to the intended users. This study is presented in Supporting Information S1 Road Testing and S2 Data.

While reporting animal research in adherence to all 21 items of ARRIVE 2.0 represents best practice, the classification of the items into two groups is intended to facilitate the improved reporting of animal research by allowing an initial focus on the most critical issues. This better allows journal staff, editors and reviewers to verify that the items have been adequately reported in manuscripts. The first step should be to ensure compliance with the ARRIVE Essential 10 as a minimum requirement. Items from the Recommended Set can then be added over time and in line with specific editorial policies until all the items are routinely reported in all manuscripts. ARRIVE 2.0 are fully compatible with and complementary to other guidelines that have been published in recent years. By providing a comprehensive set of recommendations that are specifically tailored to the description of in vivo research, they help authors reporting animal experiments adhere to the National Institutes of Health standards^43^ and the minimum standards framework and checklist (Materials, Design, Analysis and Reporting^47^). The revised guidelines are also in line with many journals’ policies and will assist authors in complying with information requirements on the ethical review of the research,^48 49^ data presentation and access,^50–52^ statistical methods^51 52^ and conflicts of interest.^53 54^

Although the guidelines are written with researchers and journal editorial policies in mind, it is important to stress that researchers alone should not have to carry the responsibility for transparent reporting. Funders’, institutions’ and publishers’ endorsement of ARRIVE has been instrumental in raising awareness to date; they now have a key role to play in building capacity and championing the behavioural changes required to improve reporting practices. This includes embedding ARRIVE 2.0 in appropriate training, workflows and processes to support researchers in their different roles. While the primary focus of the guidelines has been on the reporting of animal studies, ARRIVE also has other applications earlier in the research process, including in the planning and design of in vivo experiments. For example, requesting a description of the study design in line with the guidelines in funding or ethical review applications ensures that steps to minimise experimental bias are considered at the beginning of the research cycle.^55^

## Conclusion

Transparent reporting is clearly essential if animal studies are to add to the knowledge base and inform future research, policy and clinical practice. ARRIVE 2.0 prioritises the reporting of information related to study reliability. This enables research users to assess how much weight to ascribe to the findings and, in parallel, promotes the use of rigorous methodology in the planning and conduct of in vivo experiments,^37^ thus increasing the likelihood that the findings are reliable and, ultimately, reproducible.

The intention of ARRIVE 2.0 is not to supersede individual journal requirements but to promote a harmonised approach across journals to ensure that all manuscripts contain the essential information needed to appraise the research. Journals usually share a common objective of improving the methodological rigour and reproducibility of the research they publish, but different journals emphasise different pieces of information.^56–58^ Here, we propose an expert consensus on information to prioritise. This will provide clarity for authors, facilitate transfer of manuscripts between journals, and accelerate an improvement of reporting standards.

Concentrating the efforts of the research and publishing communities on the ARRIVE Essential 10 items provides a manageable approach to evaluate reporting quality efficiently and assess the effect of interventions and policies designed to improve the reporting of animal experiments. It provides a starting point for the development of operationalised checklists to assess reporting, ultimately leading to the build of automated or semi-automated artificial intelligence tools that can detect missing information rapidly.^59^

Improving reporting is a collaborative endeavour, and concerted effort from the biomedical research community is required to ensure maximum impact. We welcome collaboration with other groups operating in this area, as well as feedback on ARRIVE 2.0 and our implementation strategy.
